# Visceral adipose tissue in the lesser omentum predicts lymphovascular invasion, perineural invasion and survival in gastric cancer

**DOI:** 10.3389/fonc.2025.1555824

**Published:** 2025-06-19

**Authors:** Ping-ping Liu, Le Liu, Han-bing Xie, Lin Zhao, Shuo Pang, Rui-han Zhou, Shu-rui Wang, Shi-di Miao, Rui-tao Wang, Shuang Fu

**Affiliations:** ^1^ Department of Internal Medicine, Harbin Medical University Cancer Hospital, Harbin Medical University, Harbin, Heilongjiang, China; ^2^ School of Computer Science and Technology, Harbin University of Science and Technology, Harbin, Heilongjiang, China

**Keywords:** gastric cancer, visceral adipose tissue, prognosis, lymphovascular invasion, perineural invasion

## Abstract

**Background:**

Visceral adipose tissue is associated with clinical outcomes in patients with cancer. This study aimed to investigate the relationship between preoperative visceral adipose tissue in the lesser omentum and clinical prognosis, as well as lymphovascular invasion (LVI) and perineural invasion (PNI), in patients with gastric cancer (GC).

**Patients and methods:**

A total of 943 GC patients who underwent radical surgery across three centers in China were included in the study. The patients were divided into one main cohort (center 1) consisting of 389 cases for the primary set and 165 cases for the internal validation set, as well as two external validation cohorts. Preoperative visceral fat area (VFA) in the lesser omentum was measured through radiological assessments using standard computed tomography. Survival analysis was conducted using Kaplan-Meier plots and Cox proportional risk regression models. Additionally, logistic regression analysis was utilized to identify independent risk factors for LVI and PNI in GC.

**Results:**

Patients with low VFA in the lesser omentum (VFA-lesser omentum) exhibited shorter overall survival compared to those with high VFA-lesser omentum [training set: hazard ratio 0.791, 95% CI 0.665-0.941, p = 0.008; validation set: hazard ratio 0.882, 95% CI 0.792-0.982, p = 0.022]. Furthermore, reduced VFA-lesser omentum was an independent risk factor for LVI (odds ratio [OR] 0.917, 95% CI 0.860-0.978, p = 0.008) and PNI (OR 0.933, 95% CI 0.878-0.990, p = 0.023). The results were confirmed in the internal and external validation sets (both p < 0.05).

**Conclusion:**

Preoperative VFA-lesser omentum was associated with PNI and LVI. In addition, reduced VFA-lesser omentum predicts poor survival in GC patients.

## Introduction

Gastric cancer (GC) ranks as the fifth most diagnosed cancer globally and the third leading cause of cancer-related deaths (1). Radical gastrectomy is currently the most effective treatment for GC, yet the disease's high propensity for metastasis and recurrence after surgery contributes to its poor prognosis (2). The lack of effective and relevant prognostic indicators is one of the reasons for the poor prognosis of GC (3).

The correlation between obesity and GC prognosis has been controversial (4). Previous studies have shown that obese GC patients have better long-term survival than non-overweight/obese patients (5, 6). However, several studies have shown that obesity is not associated with survival in GC (7–9). Body mass index (BMI) is commonly used to assess obesity in most studies because of its convenience and objectivity, but it may not accurately reflect individual fat accumulation and body composition differences (10). Visceral adipose tissue is considered a better indicator of adipocyte dysfunction (11, 12). Recently, several studies have found that low visceral fat is an independent risk factor for poor compliance with adjuvant chemotherapy and a poor prognostic factor for survival after radical gastrectomy in GC patients (13–15).

The "gold standard" for measuring visceral fat is quantitative radiological measurement using standard computed tomography (CT) scans (16). Previous studies on visceral fat have mainly focused on the horizontal plane of L3 (the third lumbar vertebra) or the umbilicus (17). While some studies suggest visceral fat at L3 correlates with systemic metabolic risk (18), others argue region-specific visceral fat (e.g., epicardial or mesenteric fat) better reflects local pathological interactions (19–21). However, visceral fat at the L3 level does not reflect changes in the local tumor microenvironment in GC. The lesser omentum is a dual-layered membrane structure that connects the hepatic hilum and the gastric lesser curvature, containing important anatomical structures like the lymph nodes, blood vessels, and nerves. The presence of the lesser omental capsule affects the segregation and flow of fluids in the peritoneal cavity, so tumor implantation and metastasis tend to occur in this area (22). Given its anatomical proximity to gastric lymphatic and neural networks, we hypothesize that lesser omental visceral fat may directly facilitate LVI and PNI—established poor prognostic factors—through mechanical compression or adipokine-mediated signaling, thereby influencing survival.

The relationship between preoperative visceral fat in the lesser omentum and the lymphovascular invasion (LVI), perineural invasion (PNI), and overall survival (OS) in GC was not investigated.

## Patients and methods

### Study population

**Figure 1:**
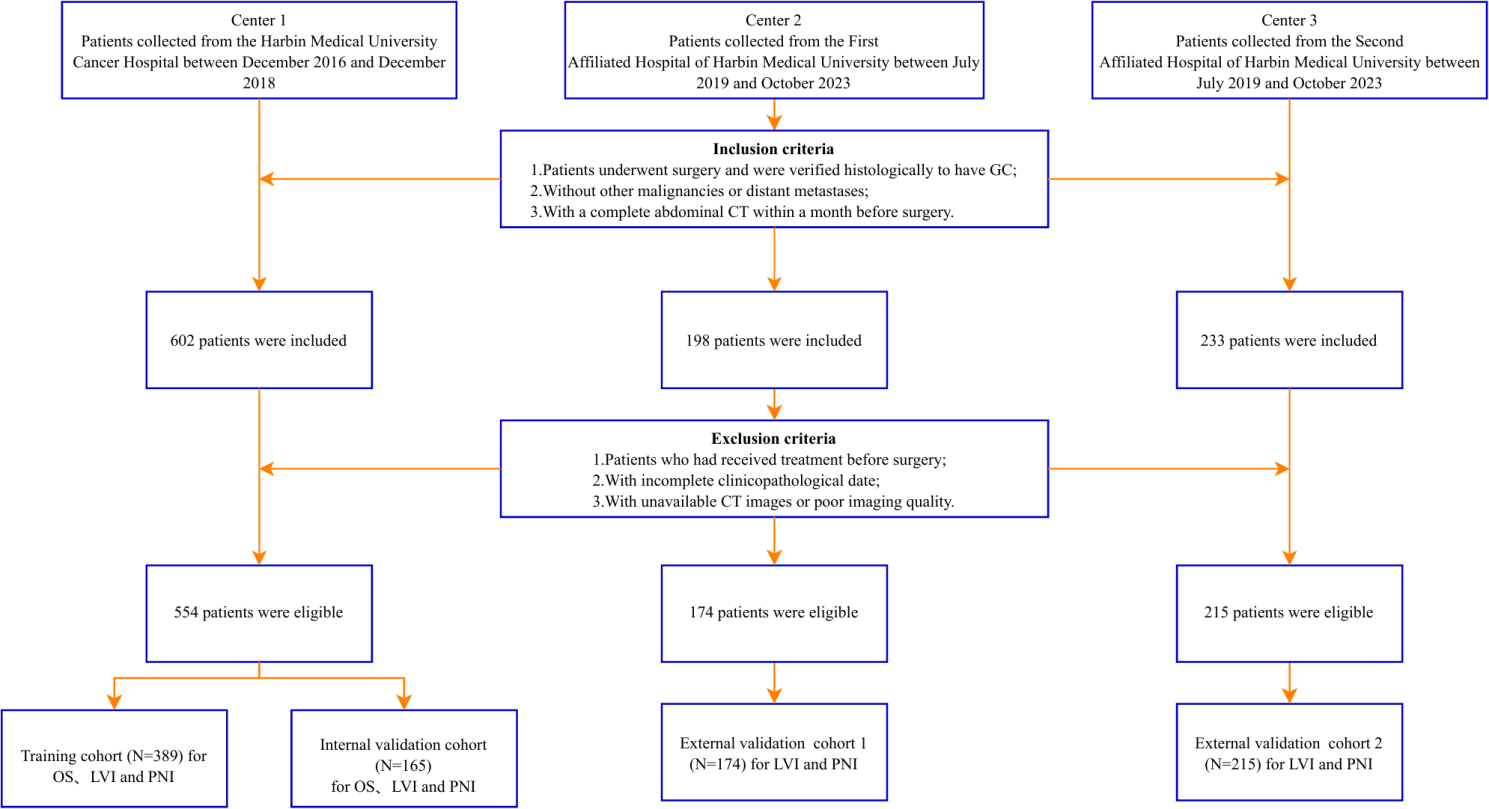
Flow diagram of the study population. GC, gastric cancer; OS, overall survival; LVI, lymphovascular invasion; PNI, perineural invasion.

This retrospective study was a multicenter study involving 943 patients from three hospitals who underwent radical surgery. The primary cohort consisted of 554 GC patients from Centre 1 (Harbin Medical University Cancer Hospital) between December 2016 and December 2018, who were randomly assigned to a training set (n=389) and an internal validation set (n=165) in a 7:3 ratio. The two external validation cohorts comprised 174 patients from Centre 2 (the Second Hospital of Harbin Medical University) between July 2019 and October 2023 and 215 patients from Centre 3 (the First Hospital of Harbin Medical University) between July 2019 and October 2023. Clinical and laboratory data, including medical records and images, were collected using the hospital's electronic medical record system. Three factors were considered as inclusion criteria (1): Patients underwent surgery and were verified histologically to have GC (2); without other malignancies or distant metastases; and (3) with a complete abdominal CT within a month before surgery. The following patients were excluded: (1) patients who had received treatment before surgery; (2) with incomplete clinicopathological data; and (3) with unavailable CT images or poor imaging quality. The procedures for the enrollment of patients are shown in **Figure 1**.

All patients were followed up every three months for the first two years and every six months during years three to five post-operation. Overall survival (OS) was defined as the duration from the date of surgery to the date of either death or the last follow-up. The median follow-up period for the present cohort was 60 months, and follow-up ended in December 2023. The study adhered to the Declaration of Helsinki guidelines and was approved by the institutional review boards of three centers under authorization number KY2024-58. Due to the retrospective nature of the study, the requirement for patient-informed consent was waived.

### Data collection

Patients' clinical data, such as age, sex, body mass index (BMI), tumor location, tumor size, differentiation, histological type, lymph node metastasis, LVI, and PNI, were collected from the electronic medical records. GC patients were staged following the guidelines of the American Joint Committee on Cancer (AJCC) staging manual (8th edition) (23).

Pathological tissue sections from GC patients were fixed and paraffin-embedded. Routine hematoxylin-eosin (HE) staining and pathological examination were performed. Tumor cells that encircled the nerve surface, infiltrated the nerve periphery, or penetrated the nerve fascia were classified as PNI (24). Additionally, the presence of at least one cluster of tumor cells within lymphatic vessels or blood vessels was defined as LVI (25). All resected specimens were histologically examined by two experienced pathologists blinded to the clinical data. In cases of disagreement, a third pathologist was consulted to reach a consensus. Cohen's kappa values for inter-observer agreement were calculated for key pathological features, yielding κ=0.85 for PNI and κ=0.78 for LVI, indicating substantial to almost perfect agreement.

### Measurement of VFA

**Figure 2:**
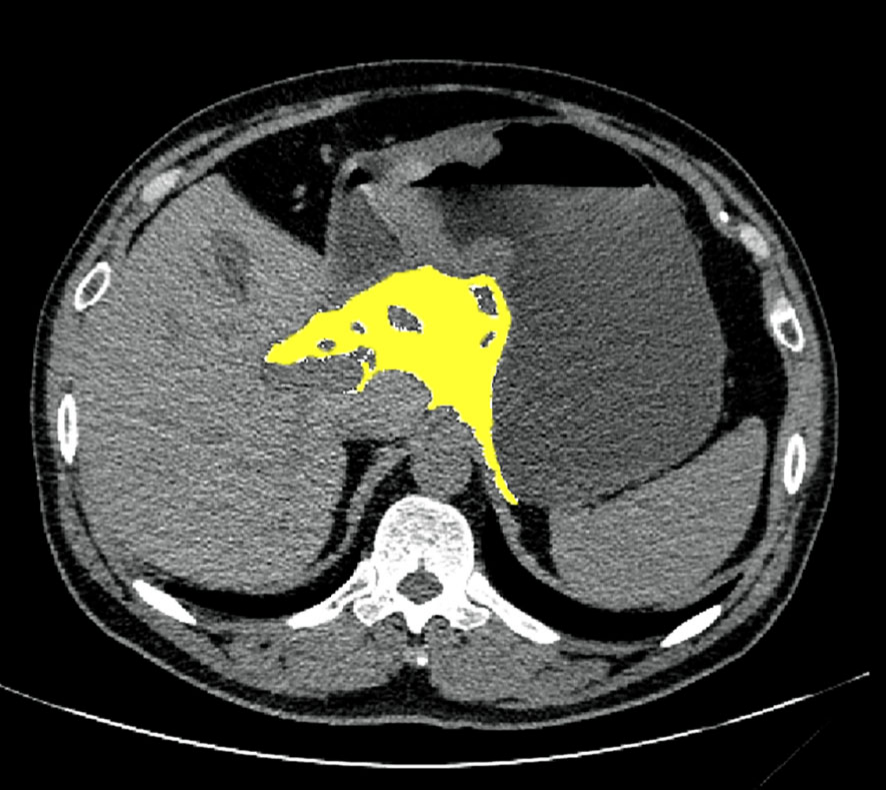
Yellow shading represents visceral fat areas in the lesser omentum, which were identified and quantified within a range of -195 to -45 HU.

Preoperative abdominal CT images were obtained in DICOM format. Visceral fat area (VFA) measurements were performed at three anatomical levels (the largest tumor, the lesser omentum nearest the tumor, and L3 vertebra). The lesser omentum on the CT image is a double membrane structure located in the lesser curvature of the stomach interconnected with the hepatic hilum (**Figure 2**). Two blinded radiologists independently analyzed all images using ImageJ (v1.53a) with HU thresholds of -195 to -45 for visceral fat detection (26). Discrepancies >5% in VFA measurements were resolved through consensus discussion with a third senior radiologist. Inter-observer agreement was excellent (intraclass correlation coefficient=0.92, 95%CI 0.88-0.95).

### Statistical analysis

Normally distributed continuous variables were reported as means with standard deviations and were compared using two-tailed Student t-test. Categorical variables were presented as frequencies and percentages and were compared using the chi-square test or Fisher's exact test. Univariate and multivariate analyses were performed by Cox proportional hazards regression models to determine the hazard ratio of each factor. Variables with p<0.10 in univariate analysis and those clinically relevant to patients' prognosis (age and gender) were included in multivariate analysis. Hazard ratio (HR) and 95% confidence interval (95% CI) were calculated. Since no prior studies have established thresholds for VFA-lesser omentum in GC to reference, we determined the optimal cut-off value using ROC curve analysis. Survival analysis was performed using the Kaplan-Meier method to plot survival graphs and calculate survival rates, and Log-rank test was used to compare survival rates between groups. The risk factors associated with LVI and PNI were analyzed using univariate and multivariate logistic regression models, and relationships were presented in forest plots. Variables showing marginal significance (p<0.10) in univariate analysis were included in the multivariate model to avoid premature exclusion of potentially important covariates, as recommended for prognostic studies (27–29). This conservative approach helps identify variables that may achieve significance when adjusted for other factors. All reported p-values are two-sided, with p<0.05 considered statistically significant in final models. All statistical analyses were conducted using SPSS version 26.0 software (IBM, New York, USA).

## Results

### Clinical characteristics

A total of 1,312 patients who underwent radical GC surgery were reviewed, and 943 patients met the study's inclusion criteria. Of all patients, 682 (72.3%) were men and 261 (27.7%) were women, with a median age of 62 (55–68) years. The main cohort of 554 patients was randomly divided into primary and validation cohorts using a conventional 7:3 ratio, which provides adequate statistical power for model development while retaining sufficient validation samples. The clinical and pathological characteristics were analyzed based on survival status, as detailed in **Table 1**. In the primary cohort, significant factors included BMI, tumor size, differentiation, PNI, LVI, tumor location, Lauren typing, T stage, lymph node status, pathological staging, postoperative chemotherapy, Borrmann classification, carbohydrate antigen 19-9, albumin, hemoglobin, VFA in the lesser omentum (VFA-lesser omentum), VFA at maximum tumor level (VFA-maximum tumor), and VFA at L3 level (VFA-L3). Non-significant variables (all p>0.05) included: age, gender, smoking history, drinking history, hypertension, diabetes mellitus, CEA, CA724, white blood cell, and platelet count. Similar results were observed in the validation set.

**Table 1:** Baseline characteristics of patients.

Variables	Training cohort (N = 389)			Validation cohort (N = 165)		
	Survival (*N* = 219)	Death (*N* = 170)	*P* value	Survival (*N* = 107)	Death (*N* = 58)	*P* value
Age (years)	59.7 ± 9.3	59.5 ± 9.4	0.882	58.3 ± 9.8	60.0 ± 11.2	0.302
BMI (kg/m^2^)	23.3 ± 3.2	22.4 ± 3.0	0.004	23.0 ± 3.3	22.1 ± 3.4	0.086
Gender			0.458			0.163
Male	162 (74.0)	120 (70.6)		76 (71.0)	35 (60.3)	
Female	57 (26.0)	50 (29.4)		31 (29.0)	23 (39.7)	
Smoking			0.822			0.184
Yes	116 (53.0)	92 (54.1)		54 (50.5)	23 (39.7)	
No	103 (47.0)	78 (45.9)		53 (49.5)	35 (60.3)	
Drinking			0.809			0.613
Yes	76 (34.7)	57 (33.5)		31 (29.0)	19 (32.8)	
No	143 (65.3)	113 (66.5)		76 (71.0)	39 (67.2)	
Hypertension			0.760			0.771
No	183 (83.6)	144 (84.7)		99 (92.5)	53 (91.4)	
Yes	36 16.4)	26 (15.3)		8 (7.5)	5 (8.6)	
Diabetes			0.249			0.278
No	202 (92.2)	151 (88.8)		98 (91.6)	50 (86.2)	
Yes	17 (7.8)	19 (11.2)		9 (8.4)	8 (13.8)	
Tumor size (cm)			<0.001			0.001
< 4	146 (66.7)	66 (38.8)		71 (66.4)	23 (39.7)	
≥ 4	73 (33.3)	104 (61.2)		36 (33.6)	35 (60.3)	
Differentiation			0.003			<0.001
Well/Moderately	101 (46.1)	53 (31.2)		55 (51.4)	12 (20.7)	
Poorly/Undifferentiated	118 (53.9)	117 (68.8)		52 (48.6)	46 (79.3)	
Lymphovascular invasion			<0.001			0.010
Negative	142 (64.8)	60 (35.3)		65 (60.7)	23 (39.7)	
Positive	77 (35.2)	110 (64.7)		42 (39.3)	35 (60.3)	
Perineural invasion			<0.001			0.001
Negative	127 (58.0)	27 (15.9)		61 (57.0)	18 (31.0)	
Positive	92 (42.0)	143 (84.1)		46 (43.0)	40 (69.0)	
Location			0.003			1.000
Cardia	31 (14.2)	31 (18.2)		13 (12.2)	14 (24.1)	
Body	53 (24.2)	36 (21.2)		30 (28.0)	13 (22.4)	
Antrum	133 (60.7)	90 (52.9)		64 (59.8)	25 (43.1)	
Whole stomach	2 (0.9)	13 (7.7)		0 (0.0)	6 (10.4)	
Surgical procedure			0.008			<0.001
Partial gastrectomy	184 (84.0)	124 (72.9)		92 (86.0)	36 (62.1)	
Total gastrectomy	35 (16.0)	46 (27.1)		15 (14.0)	22 (37.9)	
D2 lymph node dissection			<0.001			<0.001
Yes	133 (60.7)	159 (93.5)		70 (65.4)	56 (96.6)	
No	86 (39.3)	11 (6.5)		37 (34.6)	2 (3.4)	
Lauren type			0.007			<0.001
Diffuse	70 (32.0)	70 (41.2)		31 (29.0)	27 (46.6)	
Intestinal	108 (49.3)	57 (33.5)		54 (50.4)	11 (19.0)	
Mixed	41 (18.7)	43 (25.3)		22 (20.6)	20 (34.4)	
Depth of invasion			<0.001			<0.001
T1/T2	110 (50.2)	14 (8.2)		55 (51.4)	6 (10.3)	
T3/T4	109 (49.8)	156 (91.8)		52 (48.6)	52 (89.7)	
Lymph node metastasis			<0.001			<0.001
Yes	98 (44.7)	151 (88.8)		43 (40.2)	52 (89.7)	
No	121 (55.3)	19 (11.2)		64 (59.8)	6 (10.3)	
pTNM stage			<0.001			<0.001
I/ II	168 (76.7)	45 (26.5)		83 (77.6)	11 (19.0)	
III	51 (23.3)	125 (73.5)		24 (22.4)	47 (81.0)	
Adjuvant chemotherapy			<0.001			<0.001
Yes	84 (38.4)	152 (89.4)		43 (40.2)	53 (91.4)	
No	135 (61.6)	18 (10.6)		64 (59.8)	5 (8.6)	
Borrmann type			<0.001			<0.001
Type 1	9 (4.1)	5 (2.9)		8 (7.5)	1 (1.7)	
Type 2	59 (26.9)	16 (9.4)		31 (29.0)	7 (12.1)	
Type 3	148 (67.6)	123 (72.4)		67 (62.6)	40 (69.0)	
Type 4	3 (1.4)	26 (15.3)		1 (0.9)	10 (17.2)	
CEA			0.108			0.236
Normal	193 (88.1)	140 (82.4)		97 (90.7)	49 (84.5)	
Elevated	26 (11.9)	30 (17.6)		10 (9.3)	9 (15.5)	
CA199			0.001			0.006
Normal	203 (92.7)	139 (81.8)		99 (92.5)	45 (77.6)	
Elevated	16 (7.3)	31 (18.2)		8 (7.5)	13 (22.4)	
CA724			0.151			0.331
Normal	15 (6.8)	6 (3.5)		9 (8.4)	2 (3.4)	
Elevated	204 (93.2)	164 (96.5)		98 (91.6)	56 (96.6)	
Albumin (g/L)	40.3 ± 5.1	38.8 ± 4.8	0.003	40.2 ± 4.8	38.5 ± 5.9	0.051
White blood cell (10^9 /L)	6.53 ± 2.01	6.36 ± 1.90	0.408	6.43 ± 2.01	6.89 ± 1.97	0.160
Platelet (10^9 /L)	244.9 ± 77.7	255.8 ± 82.9	0.181	241.4 ± 67.9	290.7 ± 88.2	<0.001
Hemoglobin (g/L)	134.6 ± 21.9	127.9 ± 24.2	0.005	133.4 ± 27.3	123.9 ± 23.5	0.026
VFA-lesser omentum (cm^2^)	11.28 ± 7.97	7.52 ± 5.84	<0.001	10.37 ± 7.98	6.61 ± 5.32	<0.001
VFA-tumor maximum (cm^2^)	66.60 ± 45.26	52.14 ± 40.18	0.001	64.06 ± 44.16	45.94 ± 35.86	0.007
VFA-L3 (cm^2^)	115.85 ± 65.25	99.21 ± 63.92	0.025	111.18 ± 63.89	89.67 ± 51.35	0.052

BMI, body mass index; CEA, carcinoembryonic antigen; CA199, carbohydrate antigen 19-9; CA724, carbohydrate antigen 724; VFA, visceral fat area; L3, the third lumbar vertebra.

### Predictors of survival

Cox regression analysis was conducted to determine the independent predictors of OS. In the training set, univariate analysis revealed significant associations between OS and various factors, including BMI, pathological tumor size, differentiation, LVI, PNI, tumor location, Lauren type, T-staging, lymph node status, TNM staging, postoperative chemotherapy, Borrmann classification, VFA-lesser omentum, VFA-maximum tumor, and VFA-L3. The following variables did not show significant associations with survival: age, gender, drinking history, hypertension, and diabetes mellitus. Then, factors with a p-value <0.10 in univariate analysis were included in multivariate analysis, which identified VFA-lesser omentum as an independent predictor of OS. The Cox regression analyses in the validation set confirmed this finding (**Table 2**).

**Table 2:** Univariate analysis and multivariate analysis in the training and internal validation cohort.

Variables	Univariate Analysis		Multivariate Analysis	
	Hazard ratio (95% CI)	*P*	Hazard ratio (95% CI)	*P*
Training cohort				
Age (years)	1.002 (0.985 – 1.018)	0.846		
BMI (kg/m^2^)	0.931 (0.885– 0.978)	0.005	0.996 (0.928 – 1.069)	0.915
Gender (Male vs Female)	1.120 (0.805 –1.558)	0.500		
Smoking (Yes vs No)	0.963 (0.712 – 1.302)	0.807		
Drinking (Yes vs No)	0.999 (0.727 – 1.374)	0.995		
Hypertension (Yes vs No)	0.929 (0.612 – 1.411)	0.731		
Diabetes (Yes vs No)	1.193 (0.740 – 1.923)	0.468		
Tumor size (≥4cm vs <4cm)	2.287 (1.679 – 3.116)	< 0.001	1.041 (0.729 – 1.486)	0.825
Differentiation (Poorly/Undifferentiated vs Well/Moderately)	1.645 (1.189 – 2.276)	0.003	1.078 (0.714 – 1.628)	0.720
Lymphovascular invasion (Present vs Absent)	2.399 (1.750 – 3.288)	< 0.001	1.107 (0.764 – 1.604)	0.592
Perineural invasion (Present vs Absent)	4.932 (3.264 – 7.451)	< 0.001	2.044 (1.232 – 3.393)	**0.006**
Tumor location		0.003		0.833
Cardia	Ref		Ref	
Body	0.756 (0.468 – 1.222)	0.254	0.989 (0.397 – 2.463)	0.981
Antrum	0.770 (0.512 – 1.159)	0.210	0.411 (0.057 – 2.960)	0.378
Whole	2.600 (1.357 – 4.979)	0.004	0.410 (0.054 – 3.122)	0.390
Surgical procedure (Total gastrectomy vs Partial gastrectomy)	1.576 (1.123 – 2.212)	0.008	1.339 (0.053 – 2.151)	0.390
D2 lymph node dissection (Yes vs No)	0.161 (0.087 – 0.297)	< 0.001	0.905 (0.696 – 5.124)	0.210
Lauren type (Diffuse vs Intestinal/Mixed)	1.370 (1.009 – 1.860)	0.043	0.849 (0.576 – 1.254)	0.411
T classification (T3/T4 vs T1/T2)	7.462 (4.314 – 12.906)	< 0.001	2.013 (0.802 – 5.053)	0.136
Lymph node status (Yes vs No)	6.527 (4.046 – 10.531)	< 0.001	0.837 (0.294 – 2.381)	0.739
TNM stage (III/IV vs I/II)	5.379 (3.815 – 7.586)	< 0.001	1.886 (1.157 – 3.075)	**0.011**
Adjuvant chemotherapy (Yes vs No)	0.118 (0.072 – 0.193)	< 0.001	0.291 (0.087 – 0.978)	**0.046**
Borrmann type (Type 4 vs Type 1/2/3)	3.524 (2.309 – 5.377)	< 0.001	1.431 (0.789 – 2.597)	0.238
VFA-lesser omentum (cm^2^)	0.941 (0.918 – 0.964)	< 0.001	0.943 (0.906 – 0.983)	**0.005**
VFA-maximum tumor (cm^2^)	0.993 (0.989 – 0.997)	0.001	1.000 (0.993 – 1.008)	0.887
VFA-L3 (cm^2^)	0.997 (0.995 – 1.000)	0.028	1.003 (0.999 – 1.007)	0.139
Validation cohort				
Age (years)	1.019 (0.992 – 1.046)	0.170		
BMI (kg/m^2^)	0.929 (0.854– 1.010)	0.086	0.791 (0.665 – 0.941)	**0.008**
Gender (Male vs Female)	1.536 (0.907 – 2.600)	0.110		
Smoking (Yes vs No)	0.699 (0.413 – 1.183)	0.182		
Drinking (Yes vs No)	1.121 (0.648 – 1.939)	0.684		
Hypertension (Yes vs No)	1.053 (0.421 – 2.635)	0.912		
Diabetes (Yes vs No)	1.597 (0.756 – 3.370)	0.220		
Tumor size (≥4cm vs <4cm)	2.305 (1.361 – 3.903)	0.002	1.085 (0.470 – 2.502)	0.849
Differentiation (Poorly/Undifferentiated vs Well/Moderately)	3.049 (1.614 – 5.760)	0.001	0.785 (0.325 – 1.896)	0.591
Lymphovascular invasion (Present vs Absent)	2.027 (1.197 – 3.432)	0.009	0.562 (0.262 – 1.203)	0.138
Perineural invasion (Present vs Absent)	2.414 (1.383 – 4.213)	0.002	0.928 (0.411 – 2.092)	0.856
Tumor location		< 0.001		0.642
Cardia	Ref		Ref	
Body	0.505 (0.237 – 1.074)	0.076	0.858 (0.164 – 4.493)	0.857
Antrum	0.443 (0.230 – 0.853)	0.015	0.711 (0.046 – 10.976)	0.807
Whole	3.098 (1.175 – 8.165)	0.022	0.397 (0.027 – 5.739)	0.498
Surgical procedure (Total gastrectomy vs Partial gastrectomy)	2.745 (1.613 – 4.671)	< 0.001	1.119 (0.122 – 10.245)	0.921
D2 lymph node dissection (Yes vs No)	0.092 (0.022 – 0.377)	0.001	0.265 (0.038 – 1.819)	0.177
Lauren type (Diffuse vs Intestinal/Mixed)	1.772 (1.058 – 2.970)	0.030	1.036 (0.492 – 2.181)	0.925
T classification (T3/T4 vs T1/T2)	6.489 (2.785 – 15.123)	< 0.001	0.385 (0.062 – 2.399)	0.306
Lymph node status (Yes vs No)	8.504 (3.647 – 19.826)	< 0.001	0.195 (0.014 – 2.741)	0.226
TNM stage (III/IV vs I/II)	7.889 (4.077 – 15.264)	< 0.001	4.196 (1.020 – 17.261)	**0.047**
Adjuvant chemotherapy (Yes vs No)	0.097 (0.039 – 0.244)	< 0.001	0.067 (0.004 – 1.112)	0.059
Borrmann type (Type 4 vs Type 1/2/3)	4.210 (2.116 – 8.377)	< 0.001	0.867 (0.209 – 3.596)	0.844
VFA-lesser omentum (cm^2^)	0.936 (0.897 – 0.977)	0.003	0.882 (0.792 – 0.982)	**0.022**
VFA-maximum tumor (cm^2^)	0.991 (0.983 – 0.998)	0.014	1.018 (0.998 – 1.038)	0.081
VFA-L3 (cm^2^)	0.996 (0.991 – 1.000)	0.078	1.003 (0.992 – 1.014)	0.618

BMI, body mass index; VFA, visceral fat area; L3, the third lumbar vertebra. Bold values represent statistically significant differences (P< 0.05).

**Figure 3:**
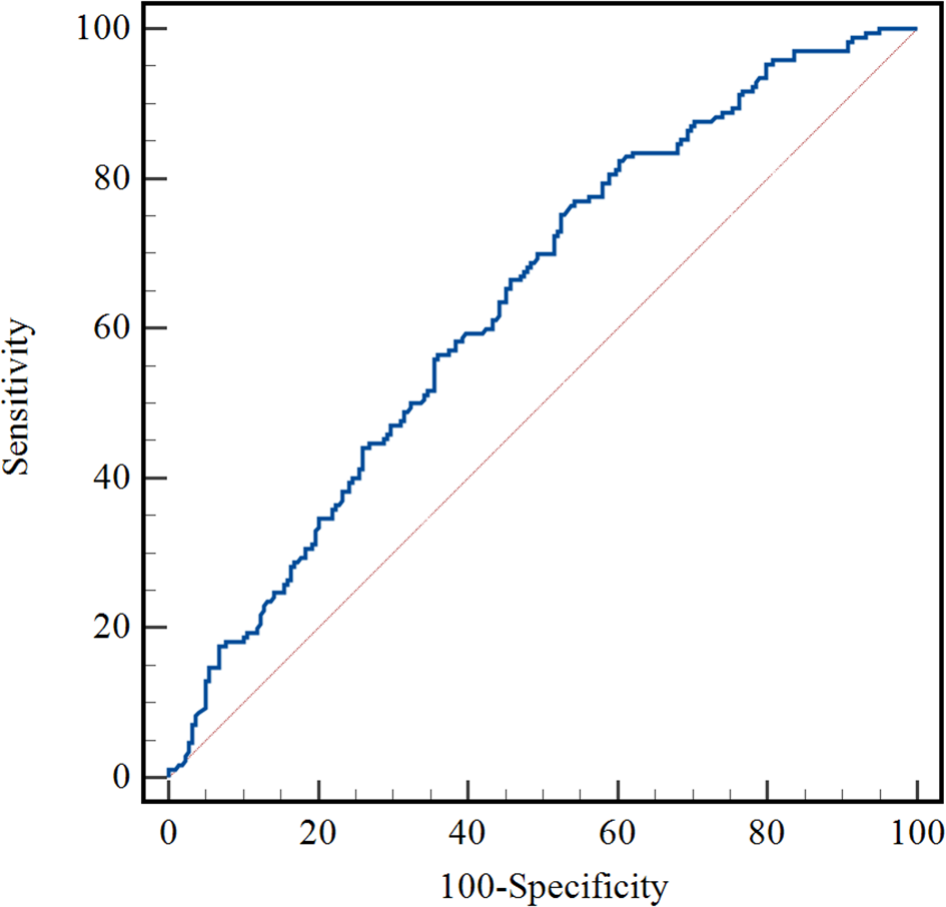
An optimized cut-off value was determined for VFA in lesser omentum using ROC curve analysis. VFA, visceral fat area; ROC curve, receiver operating characteristic curve.

**Figure 4:**
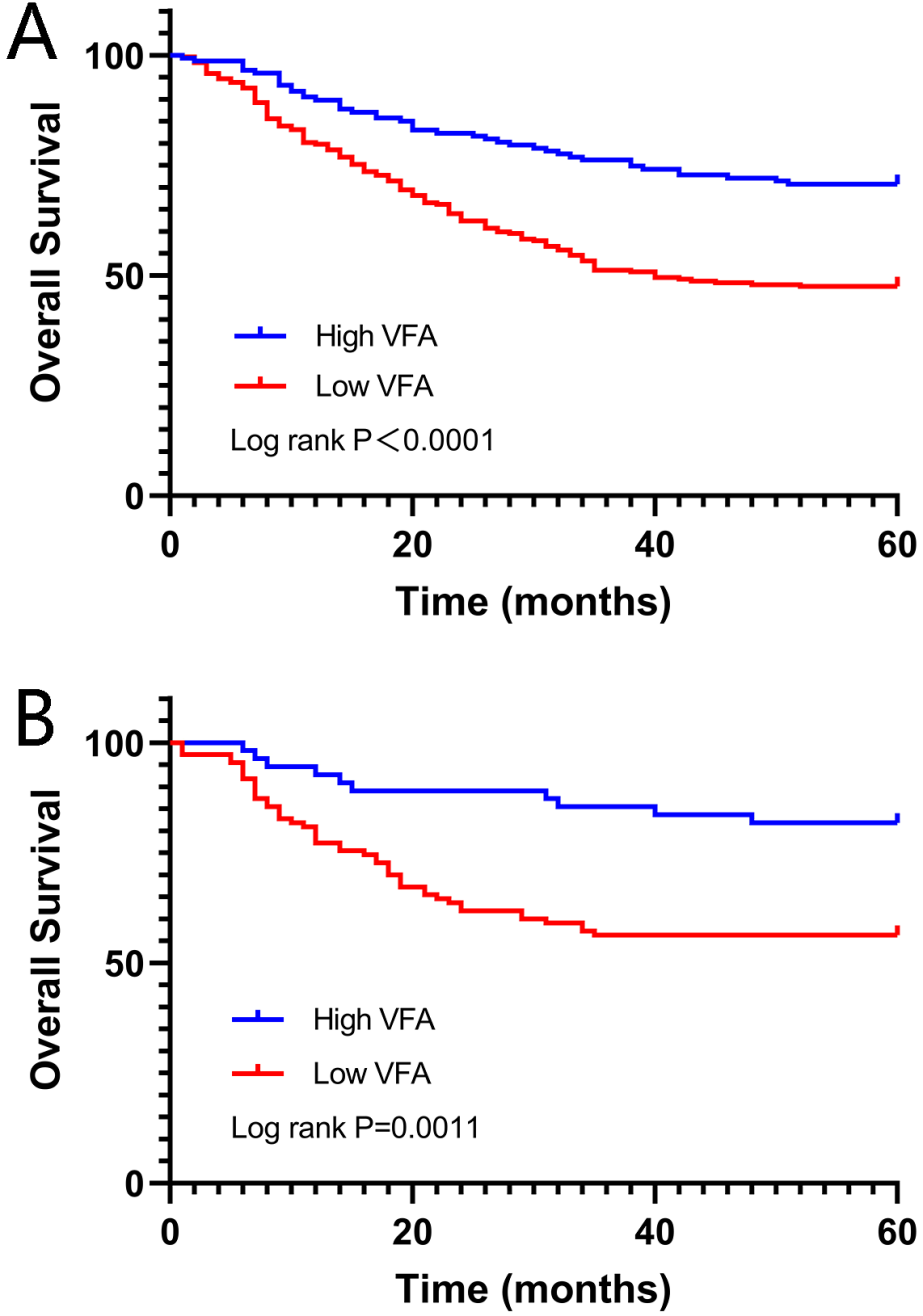
Kaplan-Meier analysis of overall survival in gastric cancer patients according to VFA in lesser omentum. **(A)** training set; **(B)** validation set.

The optimal cut-off value of VFA-lesser omentum was determined by receiver operating characteristics (ROC) curve analysis, dividing patients into high-VFA group (VFA ≥ 11.13cm^2^) and low-VFA group (VFA < 11.13cm^2^), with a sensitivity and specificity of 75.3% and 47.5%, respectively (**Figure 3**). Of the 389 patients in the training set, 170 died during follow-up, 43 patients in the high-VFA group, and 127 patients in the low-VFA group. Patients in the low group had shorter OS than those in the high VFA group (38.19 months vs. 48.72 months, log-rank p < 0.0001; **Figure 4A**). Similarly, in the validation set, VFA-lesser omentum in the low versus high group showed a significant separation of the Kaplan-Meier OS curves (log-rank p=0.0011; **Figure 4B**).

### Association of VFA-lesser omentum with LVI

**Figure 5:**
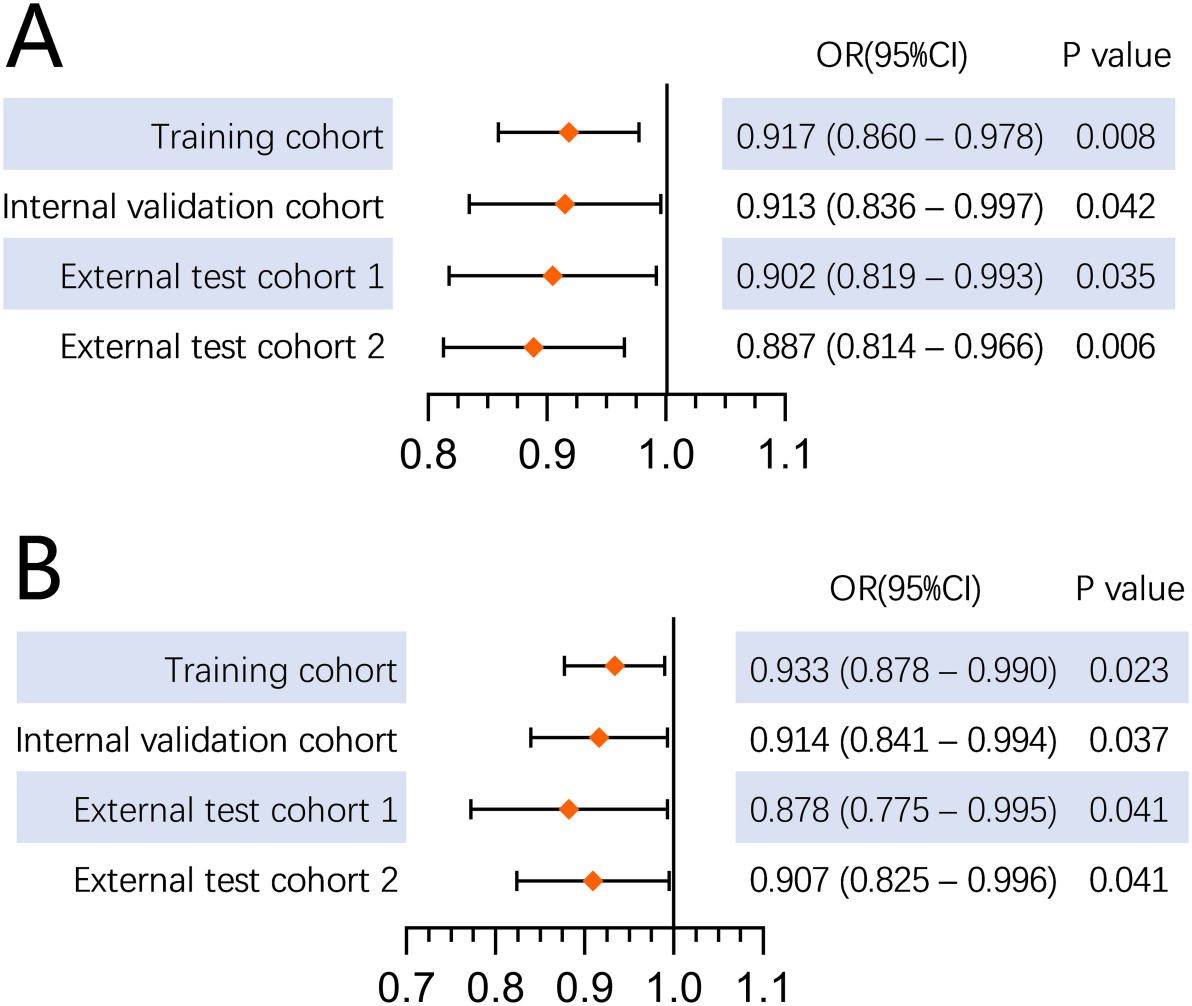
**(A)** Forest plot of the VFA in lesser omentum on LVI analysis; **(B)** Forest plot of the VFA in lesser omentum on PNI analysis.

The clinicopathological characteristics of the patients in four cohorts were summarized based on LVI status. Significant differences were found in PNI, T stage, lymph node status, TNM stage, VFA-lesser omentum, and VFA-maximum tumor (**Supplementary Table S1**). In the training set, univariate logistic regression analysis showed that BMI, pathological tumor size, differentiation, PNI, T stage, lymph node status, TNM stage, VFA-lesser omentum, VFA-maximum tumor and VFA-L3 were associated with LVI. The following variables did not show significant associations with LVI: age, gender, diabetes mellitus, tumor location, and Lauren type. Multivariate logistic regression analysis revealed that pathological tumor size, PNI, lymph node status, and VFA-lesser omentum were the independent risk factors for LVI. The other three sets confirmed that VFA-lesser omentum was an independent risk factor for LVI (**Supplementary Table S3**). A forest plot was generated showing that high VFA-lesser omentum was an independent factor for LVI (**Figure 5A**).

### Association of VFA-lesser omentum with PNI

The patients were categorized into two groups based on the presence of PNI. Significant differences were observed in LVI, T-staging, lymph node status, TNM stage, VFA-lesser omentum, and VFA-maximum tumor across the four cohorts (**Supplementary Table S2**). According to multivariate analysis, the preoperative VFA-lesser omentum was an independent risk factor for PNI (p<0.05, **Supplementary Table S4**). A forest plot illustrated the independent role of VFA-lesser omentum in the four cohorts (**Figure 5B**).

## Discussion

This study showed the value of VFA-lesser omentum in predicting the prognosis of patients with GC. In addition, the findings revealed that VFA-lesser omentum was associated with LVI and PNI. These results suggest that VFA-lesser omentum could serve as a potential biomarker for identifying patients at risk of unfavorable clinical outcomes.

The lesser omentum is a double membrane structure located in the lesser curvature of the stomach interconnected with the hepatic hilum. There will be significant adipose tissue in the lesser omentum, mainly in the form of fan-shaped or triangular fat-dense shadows. Moreover, the lesser omentum is rich in gastric blood supply, nerves, and lymphatic vessels. Lymphatic metastasis of GC often occurs in the perigastric lymph nodes of the lesser curvature of the stomach, and lymph node metastasis has been experimentally proven to be an independent risk factor for the prognosis of GC (30). The clinical impact of visceral fat at the L3 level on survival in patients with GC undergoing radical treatment has been extensively researched in the past. However, the L3 level mostly represents systemic fat distribution (31). Compared to visceral fat at the L3 level, visceral fat in the lesser omentum is specific and reflects changes in the local tumor microenvironment in GC.

The association of low VFA with a poor prognosis in GC may be related to the following: Firstly, patients with a larger VFA have better nutritional reserves to meet the demands of postoperative recovery (32). In contrast, patients with a small VFA area were unable to meet their physical needs after surgery and had a poorer prognosis. Secondly, obese patients may also have tumors that are sensitive to chemotherapy. Campbell et al. found that overweight and obese patients were more likely to have microsatellite instability tumors than normal weight patients (33). Emerging evidence in GC suggests that microsatellite instability-high (MSI-H) tumors are more likely to benefit from platinum-fluorouracil combination chemotherapy (e.g., oxaliplatin plus fluorouracil) compared to microsatellite stable (MSS) tumors, as demonstrated in recent clinical studies (34, 35). While MSI-high tumors show improved survival due to their immunogenic phenotype, our study suggests that visceral adipose tissue may independently modulate prognosis through mechanisms such as chronic inflammation or adipokine signaling. Thirdly, a late symptom of upper gastrointestinal tumors is impaired oral intake, leading to failure to absorb nutrients and subsequent persistent postoperative weight loss (36, 37). Studies have shown that a higher rate of postoperative weight loss is correlated with poorer adherence to postoperative adjuvant chemotherapy (38) and lower survival rates in GC patients (39–41). Importantly, the prognostic value of body composition extends beyond visceral fat; for example, sarcopenia (low muscle mass) has been linked to reduced survival in colorectal cancer, as shown in the study association between muscle mass and overall survival among colorectal cancer patients at tertiary cancer center in the Middle East (42), underscoring the systemic impact of body composition on gastrointestinal cancer outcomes.

This study observed that VFA in the lesser omentum was associated with LVI and PNI, likely due to its dense lymphatic and neural infrastructure facilitating local invasion. While LVI/PNI were primary endpoints (aligned with the lesser omentum’s anatomy), *post-hoc* analysis revealed unexpectedly higher LNM rates in the low-VFA group (68.6% vs. 56.5%, p=0.016; **Supplementary Table S5**), suggesting systemic mediators (e.g., adipokines (43) or inflammation (44) may indirectly drive nodal spread. The paradoxical link between low VFA and aggressive phenotypes (elevated PNI/LVI) could reflect metabolic stress in adipose-scarce microenvironments, though obesity-associated survival benefits (45) underscore the complexity of adipose-tumor interplay. Future work should delineate VFA’s impact on LNM via mechanical (lymphatic compression) vs. biochemical (cytokine/chemokine) pathways, leveraging imaging or functional assays.

Limitations of this study include: firstly, the results of this study are limited by its retrospective nature and may not be immune to the adverse effects of selection bias. Secondly, our study did not account for physical activity levels or comorbid metabolic disorders, which may confound the relationship between visceral fat and prognosis. Future studies should incorporate these variables to better isolate the role of adipose biology. Thirdly, differences in body size between races must be taken into account. As Asians tend to have a lower BMI and are less prone to obesity than Europeans, this may have influenced our results. Further studies outside of Asia are needed.

In conclusion, preoperative VFA-lesser omentum was associated with PNI and LVI. In addition, reduced VFA-lesser omentum predicts poor survival in GC patients. This study suggests the potential application of VFA in the lesser omentum for risk stratification in preoperative patients.

## Data Availability

The original contributions presented in the study are included in the article/**Supplementary Material**. Further inquiries can be directed to the corresponding author.
