# Supplementary material for: Visceral adipose tissue in the lesser omentum predicts lymphovascular invasion, perineural invasion and survival in gastric cancer
Source: Front Oncol. 2025 Jun 19;15:1555824. doi: 10.3389/fonc.2025.1555824 (PMC12221911; doi:10.3389/fonc.2025.1555824)
Supplement: Supplementary file 1 [file Table1.docx]

**Table S1**. Characteristics of gastric cancer patients with or without LVI.

| **Variables** | **Training cohort**  **(N = 389)** | | | **Internal validation cohort**  **(N = 165)** | | | **External test cohort 1**  **(N = 174)** | | | **External test cohort 2**  **(N = 215)** | | |
| --- | --- | --- | --- | --- | --- | --- | --- | --- | --- | --- | --- | --- |
|  | **LVI (–)**  **(*N* = 197)** | **LVI (+)**  **(*N* = 192)** | ***P* value** | **LVI (-)**  **(*N* = 93)** | **LVI (+)**  **(*N* = 72)** | ***P* value** | **LVI (-)**  **(*N* = 102)** | **LVI (+)**  **(*N* = 72)** | ***P* value** | **LVI (-)**  **(*N* = 65)** | **LVI (+)**  **(*N* = 150)** | ***P* value** |
| Age (years) | 59.1±9.7 | 59.6±9.0 | 0.586 | 58.9±10.3 | 60.2±10.2 | 0.441 | 63.1±8.3 | 64.1±8.7 | 0.425 | 62.2±9.0 | 62.6±9.7 | 0.746 |
| BMI (kg/m^2^) | 23.2±3.1 | 22.4±3.2 | 0.011 | 23.1±3.3 | 22.3±3.2 | 0.105 | 23.4±3.1 | 22.4±4.2 | 0.078 | 23.6±2.9 | 22.2±3.0 | 0.010 |
| Gender |  |  | 0.869 |  |  | 0.523 |  |  | 0.560 |  |  | 0.876 |
| Male | 137 (69.5) | 135 (70.3) |  | 70 (75.3) | 51 (70.8) |  | 79 (77.5) | 53 (73.6) |  | 18 (27.7) | 40 (26.7) |  |
| Female | 60 (30.5) | 57 (29.7) |  | 23 (24.7) | 21 (29.2) |  | 23 (22.5) | 19 (26.4) |  | 47 (72.3) | 110 (73.3) |  |
| Diabetes |  |  | 0.560 |  |  | 0.644 |  |  | 0.495 |  |  | 0.408 |
| No | 178 (90.4) | 170 (88.5) |  | 87 (93.5) | 66 (91.7) |  | 95 (93.1) | 65 (90.3) |  | 54 (83.1) | 131 (87.3) |  |
| Yes | 19 (9.6) | 22 (11.5) |  | 6 (6.5) | 6 (8.3) |  | 7 (6.9) | 7 (9.7) |  | 11 (16.9) | 19 (12.7) |  |
| Tumor size (cm) |  |  | < 0.001 |  |  | 0.450 |  |  | 0.003 |  |  | 0.013 |
| < 4 | 139 (70.6) | 79 (41.1) |  | 52 (55.9) | 36 (50.0) |  | 66 (64.7) | 30 (42.3) |  | 37 (56.9) | 58 (38.7) |  |
| ≥ 4 | 58 (29.4) | 113 (58.9) |  | 41 (44.1) | 36 (50.0) |  | 36 (35.3) | 41 (57.7) |  | 28 (43.1) | 92 (61.3) |  |
| Differentiation |  |  | 0.036 |  |  | 0.030 |  |  | 0.472 |  |  | 0.611 |
| Well/Moderately | 85 (43.1) | 63 (32.8) |  | 48 (51.6) | 25 (34.7) |  | 65 (63.7) | 42 (58.3) |  | 41 (63.1) | 100 (66.7) |  |
| Poorly/Undifferentiated | 112 (56.9) | 129 (67.2) |  | 45 (48.4) | 47 (65.3) |  | 37 (36.3) | 30 (41.7) |  | 24 (36.9) | 50 (33.3) |  |
| Perineural invasion |  |  | < 0.001 |  |  | 0.230 |  |  | 0.007 |  |  | < 0.001 |
| Negative | 117 (59.4) | 41 (21.4) |  | 57 (61.3) | 18 (25.0) |  | 89 (87.3) | 51 (70.8) |  | 50 (76.9) | 65 (43.3) |  |
| Positive | 80 (40.6) | 151 (78.6) |  | 36 (38.7) | 54 (75.0) |  | 13 (12.7) | 21 (29.2) |  | 15 (23.1) | 85 (56.7) |  |
| Location |  |  | 0.834 |  |  | 0.451 |  |  | 0.008 |  |  | 0.068 |
| Cardia | 29 (14.7) | 34 (17.7) |  | 13 (14.0) | 11 (15.3) |  | 4 (3.9) | 11 (15.3) |  | 2 (3.1) | 13 (8.7) |  |
| Body | 55 (27.9) | 49 (25.5) |  | 20 (21.5) | 10 (13.9) |  | 45 (44.1) | 23 (31.9) |  | 20 (30.8) | 64 (42.7) |  |
| Antrum | 106 (53.8) | 101 (52.6) |  | 58 (62.4) | 47 (65.3) |  | 51 (50.0) | 32 (44.5) |  | 41 (63.1) | 66 (44.0) |  |
| Whole stomach | 7 (3.6) | 8 (4.2) |  | 2 (2.1) | 4 (5.5) |  | 2 (2.0) | 6 (8.3) |  | 2 (3.0) | 7 (4.6) |  |
| Lauren type |  |  | 0.244 |  |  | 0.280 |  |  | 0.906 |  |  | 0.697 |
| Diffuse | 75 (38.1) | 71 (37.0) |  | 26 (28.0) | 26 (36.1) |  | 39 (38.2) | 28 (38.9) |  | 22 (33.9) | 44 (29.3) |  |
| Intestinal | 86 (43.6) | 73 (38.0) |  | 45 (48.4) | 26 (36.1) |  | 27 (26.5) | 17 (23.6) |  | 35 (53.8) | 82 (54.7) |  |
| Mixed | 36 (18.3) | 48 (25.0) |  | 22 (23.6) | 20 (27.8) |  | 36 (35.3) | 27 (37.5) |  | 8 (12.3) | 24 (16.0) |  |
| Depth of invasion |  |  | < 0.001 |  |  | < 0.001 |  |  | < 0.001 |  |  | 0.335 |
| T1/T2 | 94 (47.7) | 30 (15.6) |  | 50 (53.8) | 11 (15.3) |  | 50 (49.0) | 13 (18.1) |  | 22 (33.8) | 41(27.3) |  |
| T3/T4 | 103 (52.3) | 162 (84.4) |  | 43 (46.2) | 61 (84.7) |  | 52 (51.0) | 59 (81.9) |  | 43 (66.2) | 109 (2.7) |  |
| Lymph node metastasis |  |  | < 0.001 |  |  | < 0.001 |  |  | < 0.001 |  |  | < 0.001 |
| Yes | 90 (45.7) | 161 (83.9) |  | 36 (38.7) | 57 (79.2) |  | 45 (44.1) | 59 (81.9) |  | 28 (43.1) | 107 (71.3) |  |
| No | 107 (54.3) | 31 (16.1) |  | 57 (61.3) | 15 (20.8) |  | 57 (55.9) | 13 (18.1) |  | 37 (56.9) | 43 (28.7) |  |
| pTNM stage |  |  | < 0.001 |  |  | < 0.001 |  |  | < 0.001 |  |  | 0.059 |
| I/ II | 139 (70.6) | 69 (35.9) |  | 72 (77.4) | 27 (37.5) |  | 73 (71.6) | 23 (31.9) |  | 42 (64.6) | 76 (50.7) |  |
| III | 58 (29.4) | 123 (64.1) |  | 21 (22.6) | 45 (62.5) |  | 29 (28.4) | 49 (68.1) |  | 23 (35.4) | 74 (49.3) |  |
| CEA (ng/mL) | 8.0±35.0 | 6.1±21.3 | 0.515 | 3.3±3.7 | 6.9±20.4 | 0.135 | 5.7±18.3 | 4.8±8.0 | 0.725 | 15.2±6.9 | 6.9±20.4 | 0.300 |
| CA199 (U/mL) | 27.4±92.3 | 44.2±122.1 | 0.128 | 33.2±131.5 | 45.0±103.0 | 0.531 | 18.4±28.4 | 76.3±193.9 | 0.025 | 30.3±73.1 | 56.76±147.5 | 0.254 |
| CA724 (U/mL) | 8.1±32.8 | 8.9±29.0 | 0.795 | 5.3±11.3 | 14.3±45.1 | 0.101 | 2.3±2.3 | 4.9±5.7 | 0.078 | 1.3±0.4 | 6.9±9.6 | 0.329 |
| Albumin (g/L) | 40.2±4.8 | 39.0±5.4 | 0.022 | 39.9±4.3 | 39.3±5.8 | 0.443 | 39.1±4.3 | 36.8±5.1 | 0.002 | 40.8±4.6 | 40.9±4.5 | 0.867 |
| White blood cell (**10^9** /L) | 6.5±2.0 | 6.5±1.8 | 0.870 | 6.5±2.0 | 6.6±2.3 | 0.757 | 6.4±3.4 | 6.6±2.8 | 0.705 | 7.5±6.2 | 6.2±1.9 | 0.098 |
| Platelet (**10^9** /L) | 239.4±72.6 | 260.2±80.3 | 0.008 | 257.9±83.4 | 259.6±89.4 | 0.903 | 258.4±87.0 | 267.7±97.0 | 0.509 | 247.6±90.9 | 256.5±80.2 | 0.474 |
| Hemoglobin (g/L) | 134.2±22.2 | 128.6±25.7 | 0.022 | 132.0±23.7 | 128.8±24.8 | 0.391 | 130.7±28.5 | 118.7±29.6 | 0.008 | 128.4±23.8 | 126.1±24.0 | 0.525 |
| VFA-lesser omentum (cm^2^) | 10.89±7.94 | 7.75±6.32 | < 0.001 | 11.61±8.08 | 7.35±5.53 | < 0.001 | 11.11±7.85 | 6.96±5.38 | < 0.001 | 16.79±8.28 | 8.36±7.22 | < 0.001 |
| VFA-maximum tumor (cm^2^) | 65.39±41.69 | 53.29±43.63 | 0.003 | 70.34±44.69 | 51.92±40.07 | 0.007 | 69.35±46.60 | 55.53±38.28 | 0.040 | 89.55±45.43 | 52.85±40.38 | < 0.001 |
| VFA-L3 (cm^2^) | 109.04±56.06 | 97.10±63.06 | 0.089 | 113.63±65.54 | 96.61±62.24 | 0.145 | 118.34±68.56 | 113.20±69.62 | 0.696 | 124.42±48.42 | 102.91±56.65 | 0.151 |

LVI, lymphovascular invasion; BMI, body mass index; CEA, carcinoembryonic antigen; CA199, carbohydrate antigen 19-9; CA724, carbohydrate antigen 724; VFA, visceral fat area; L3, the third lumbar vertebra.

**Table S2**. Characteristics of gastric cancer patients with or without PNI.

|  | | | | | | | | | | | | |
| --- | --- | --- | --- | --- | --- | --- | --- | --- | --- | --- | --- | --- |
| **Variables** | **Training cohort**  **(N = 389)** | | | **Internal validation cohort**  **(N = 165)** | | | **External test cohort 1**  **(N = 174)** | | | **External test cohort 2**  **(N = 215)** | | |
|  | **PNI (-)**  **(*N* = 163)** | **PNI (+)**  **(*N* = 226)** | ***P* value** | **PNI (-)**  **(*N* = 70)** | **PNI (+)**  **(*N* = 95)** | ***P* value** | **PNI (-)**  **(*N* = 140)** | **PNI (+)**  **(*N* = 34)** | ***P* value** | **PNI (-)**  **(*N* = 115)** | **PNI (+)**  **(*N* = 100)** | ***P* value** |
| Age (years) | 59.3±9.7 | 59.4±10.1 | 0.937 | 60.0±8.6 | 59.1±8.9 | 0.512 | 63.7±8.4 | 62.7±8.4 | 0.503 | 63.5±8.8 | 61.4±10.2 | 0.101 |
| BMI (kg/m^2^) | 23.3±2.8 | 22.3±3.1 | <0.001 | 23.4±3.7 | 23.3±3.5 | 0.805 | 23.1±3.7 | 22.4±3.1 | 0.307 | 23.61±3.02 | 21.41±2.7 | < 0.001 |
| Gender |  |  | 0.614 |  |  | 0.711 |  |  | 0.212 |  |  | 0.543 |
| Male | 113 (69.3) | 162 (71.7) |  | 49 (70.0) | 69 (72.6) |  | 109 (77.9) | 23 (67.6) |  | 33 (28.7) | 25 (25.0) |  |
| Female | 50 (30.7) | 64 (28.3) |  | 21 (30.0) | 26 (27.4) |  | 31 (22.1) | 11 (32.4) |  | 82 (71.3) | 75 (75.0) |  |
| Diabetes |  |  | 0.014 |  |  | 0.120 |  |  | 0.153 |  |  | 0.707 |
| No | 138 (84.7) | 209 (92.5) |  | 68 (97.1) | 86 (90.5) |  | 131 (93.6) | 29 (85.3) |  | 98 (85.2) | 87 (87.0) |  |
| Yes | 25 (15.3) | 17 (7.5) |  | 2 (2.9) | 9 (9.5) |  | 9 (6.4) | 5 (14.7) |  | 17 (14.8) | 13 (13.0) |  |
| Tumor size (cm) |  |  | <0.001 |  |  | 0.058 |  |  | 0.004 |  |  | 0.011 |
| < 4 | 118 (72.4) | 96 (42.5) |  | 45 (64.3) | 47 (49.5) |  | 85 (60.7) | 11 (33.3) |  | 60 (52.2) | 35 (35.0) |  |
| ≥ 4 | 45 (27.6) | 130 (57.5) |  | 25 (35.7) | 48 (50.5) |  | 55 (39.3) | 22 (66.7) |  | 55 (47.8) | 65 (65.0) |  |
| Differentiation |  |  | 0.001 |  |  | 0.054 |  |  | 0.971 |  |  | 0.867 |
| Well/Moderately | 81 (49.7) | 74 (32.7) |  | 34 (48.6) | 32 (33.7) |  | 86 (61.4) | 21 (61.8) |  | 76 (66.1) | 65 (65.0) |  |
| Poorly/Undifferentiated | 82 (50.3) | 152 (67.3) |  | 36 (51.4) | 63 (66.3) |  | 54 (38.6) | 13 (38.2) |  | 39 (339) | 35 (35.0) |  |
| lymphovascular invasion |  |  | <0.001 |  |  | <0.001 |  |  | 0.007 |  |  | < 0.001 |
| Negative | 120 (73.6) | 84 (37.2) |  | 48 (68.6) | 38 (40.0) |  | 89 (63.6) | 13 (38.2) |  | 50 (43.5) | 15 (15.0) |  |
| Positive | 43 (26.4) | 142 (62.8) |  | 22 (31.4) | 57 (60.0) |  | 51 (36.4) | 21 (61.8) |  | 65 (56.5) | 85 (85.0) |  |
| Location |  |  | 0.119 |  |  | 0.001 |  |  | 0.547 |  |  | 0.645 |
| Cardia | 25 (15.4) | 35 (15.5) |  | 5 (7.1) | 24 (25.3) |  | 13 (9.3) | 2 (5.9) |  | 6 (5.2) | 9 (9.0) |  |
| Body | 40 (24.5) | 50 (22.1) |  | 23 (32.9) | 19 (20.0) |  | 54 (38.6) | 14 (41.2) |  | 45 (39.1) | 39 (39.0) |  |
| Antrum | 97 (59.5) | 130 (57.5) |  | 41 (58.6) | 44 (46.3) |  | 68 (48.6) | 15 (44.1) |  | 60 (52.2) | 47 (47.0) |  |
| Whole stomach | 1 (0.6) | 11 (4.9) |  | 1 (1.4) | 8 (8.4) |  | 5 (3.5) | 3 (8.8) |  | 4 (3.5) | 5 (5.0) |  |
| Lauren type |  |  | <0.001 |  |  | 0.160 |  |  | 0.951 |  |  | 0.993 |
| Diffuse | 55 (33.7) | 82 (36.3) |  | 22 (31.4) | 39 (41.1) |  | 54 (38.6) | 13 (38.2) |  | 35 (30.4) | 31 (31.0) |  |
| Intestinal | 82 (50.3) | 75 (33.2) |  | 37 (52.9) | 36 (37.9) |  | 36 (25.7) | 8 (23.5) |  | 63 (54.8) | 54 (54.0) |  |
| Mixed | 26 (16.0) | 69 (30.5) |  | 11 (15.7) | 20 (21.0) |  | 50 (35.7) | 13 (38.3) |  | 17 (14.8) | 18 (15.0) |  |
| Depth of invasion |  |  | <0.001 |  |  | <0.001 |  |  | <0.001 |  |  | 0.005 |
| T1/T2 | 106 (65.0) | 29 (12.8) |  | 41 (58.6) | 9 (9.5) |  | 62 (44.3) | 1 (2.9) |  | 43 (37.4) | 20 (20.0) |  |
| T3/T4 | 57 (35.0) | 197 (35.0) |  | 29 (41.4) | 86 (90.5) |  | 78 (55.7) | 33 (97.1) |  | 72 (62.6) | 80 (80.0) |  |
| Lymph node metastasis |  |  | <0.001 |  |  | <0.001 |  |  | 0.009 |  |  | 0.002 |
| Yes | 64 (39.3) | 180 (79.6) |  | 27 (38.6) | 73 (76.8) |  | 77 (55.0) | 27 (79.4) |  | 61 (53.0) | 74 (74.0) |  |
| No | 99 (60.7) | 46 (20.4) |  | 43 (61.4) | 22 (23.2) |  | 63 (45.0) | 7 (20.6) |  | 54 (47.0) | 26 (26.0) |  |
| pTNM stage |  |  | <0.001 |  |  | <0.001 |  |  | <0.001 |  |  | <0.001 |
| I/ II | 130 (79.8) | 86 (38.1) |  | 54 (77.1) | 37 (38.9) |  | 87 (62.1) | 9 (26.5) |  | 74 (64.3) | 44 (44.0) |  |
| III | 33 (20.2) | 140 (61.9) |  | 16 (22.9) | 58 (61.1) |  | 53 (37.9) | 25 (73.5) |  | 41 (35.7) | 56 (56.0) |  |
| CEA (ng/mL) | 6.7±30.5 | 5.7±19.7 | 0.711 | 4.5±12.3 | 9.0±34.0 | 0.296 | 5.8±16.4 | 3.3±4.7 | 0.409 | 12.7±46.8 | 5.4±11.6 | 0.139 |
| CA199 (U/mL) | 22.6±94.3 | 49.4±133.9 | 0.021 | 19.3±32.9 | 42.5±115.8 | 0.067 | 24.5±40.0 | 106.8±261.7 | 0.091 | 34.4±84.7 | 64.8±165.7 | 0.160 |
| CA724 (U/mL) | 5.6±11.9 | 11.3±41.7 | 0.091 | 10.7±37.9 | 6.4±12.5 | 0.365 | 3.7±4.9 | 2.9±1.4 | 0.693 | 8.8±11.9 | 3.3±3.2 | 0.147 |
| Albumin (g/L) | 39.7±4.6 | 39.1±5.1 | 0.275 | 40.7±5.7 | 39.9±5.1 | 0.320 | 38.3±4.9 | 37.4±4.3 | 0.310 | 40.9±4.3 | 40.8±4.7 | 0.971 |
| White blood cell (**10^9** /L) | 6.6±2.0 | 6.3±1.9 | 0.186 | 6.7±2.0 | 6.6±2.1 | 0.964 | 6.4±3.3 | 6.8±2.4 | 0.551 | 7.0±4.9 | 6.1±1.8 | 0.072 |
| Platelet (**10^9** /L) | 246.8±75.0 | 260.0±81.9 | 0.114 | 243.2±83.5 | 251.5±79.9 | 0.519 | 261.7±90.7 | 264.4±94.3 | 0.877 | 253.7±85.1 | 254.0±81.9 | 0.983 |
| Hemoglobin (g/L) | 134.2±23.4 | 126.7±24.5 | 0.002 | 135.8±18.7 | 133.4±26.4 | 0.524 | 126.0±29.2 | 124.5±31.2 | 0.790 | 126.5±24.3 | 127.1±23.4 | 0.860 |
| VFA-lesser omentum (cm^2^) | 11.60±8.00 | 7.43±6.14 | <0.001 | 13.22±8.69 | 7.85±5.54 | <0.001 | 10.25±7.48 | 5.85±4.60 | <0.001 | 14.50±8.60 | 6.77±6.16 | <0.001 |
| VFA-maximum tumor (cm^2^) | 69.74±42.01 | 50.49±42.04 | <0.001 | 71.55±43.91 | 52.55±34.65 | 0.003 | 67.22±44.60 | 48.86±37.24 | 0.028 | 82.40±46.35 | 42.73±32.90 | <0.001 |
| VFA-L3 (cm^2^) | 104.70±55.45 | 94.25±59.41 | 0.131 | 121.58±63.16 | 105.97±57.37 | 0.142 | 116.58±67.45 | 111.98±74.17 | 0.758 | 119.67±52.93 | 99.44±55.96 | 0.117 |

PNI, perineural invasion; BMI, body mass index; CEA, carcinoembryonic antigen; CA199, carbohydrate antigen 19-9; CA724, carbohydrate antigen 724; VFA, visceral fat area; L3, the third lumbar vertebra.

**Table S3.** Logistic regression analysis of LVI in gastric cancer.

| **Variables** | **Univariate Analysis**  **Odds ratio (95% CI) P** | | **Multivariate Analysis**  **Odds ratio (95% CI) P** | |
| --- | --- | --- | --- | --- |
| **Training cohort** |  | |  | |
| Age (years) | 1.006 (0.985 – 1.028) | 0.585 |  |  |
| BMI (kg/m^2^) | 0.921 (0.863– 0.982) | 0.012 | 0.986 (0.871 – 1.115) | 0.816 |
| Gender (Male vs Female) | 0.964 (0.625 – 1.487) | 0.869 |  |  |
| Diabetes (Yes vs No) | 1.212 (0.634 – 2.320) | 0.561 |  |  |
| Tumor size (≥4cm vs <4cm) | 3.428 (2.252 – 5.218) | < 0.001 | 1.887 (1.037 – 3.432) | **0.038** |
| Differentiation (Poorly/Undifferentiated vs Well/Moderately) | 1.554 (1.029 – 2.348) | 0.036 | 1.510 (0.835 – 2.729) | 0.173 |
| Perineural invasion (Present vs Absent) | 5.386 (3.444 – 8.424) | < 0.001 | 2.806 (1.419 – 5.547) | **0.003** |
| Tumor location |  | 0.975 |  |  |
| Cardia | Ref |  |  |  |
| Body | 0.760 (0.406 – 1.423) | 0.391 |  |  |
| Antrum | 0.813 (0.462 – 1.431) | 0.515 |  |  |
| Whole | 0.975 (0.315 – 3.014) | 0.965 |  |  |
| Lauren type (Intestinal vs Diffuse/Mixed) | 0.954 (0.633 – 1.439) | 0.824 |  |  |
| T classification (T3/T4 vs T1/T2) | 4.928 (3.051 – 7.960) | < 0.001 | 1.117 (0.438 – 2.849) | 0.817 |
| Lymph node status (Yes vs No) | 6.175 (3.837 – 9.936) | < 0.001 | 4.798 (2.047 – 11.246) | < **0.001** |
| TNM stage (III/IV vs I/II) | 4.272 (2.792 – 6.536) | < 0.001 | 0.781 (0.363 – 1.679) | 0.527 |
| VFA-lesser omentum (cm^2^) | 0.940 (0.912 – 0.968) | < 0.001 | 0.917 (0.860 – 0.978) | **0.008** |
| VFA-maximum tumor (cm^2^) | 0.993 (0.988 – 0.998) | 0.003 | 1.008 (0.996 – 1.020) | 0.213 |
| VFA-L3 (cm^2^) | 0.997 (0.993 – 1.001) | 0.090 | 1.002 (0.993 – 1.010) | 0.710 |
| **Internal validation cohort** |  |  |  |  |
| Age (years) | 1.012 (0.982 – 1.043) | 0.439 |  |  |
| BMI (kg/m^2^) | 0.923 (0.838 – 1.017) | 0.106 |  |  |
| Gender (Male vs Female) | 1.253 (0.627 – 2.506) | 0.523 |  |  |
| Diabetes (Yes vs No) | 1.318 (0.407 – 4.272) | 0.645 |  |  |
| Tumor size (≥4cm vs <4cm) | 1.268 (0.684 – 2.351) | 0.450 |  |  |
| Differentiation (Poorly/Undifferentiated vs Well/Moderately) | 2.005 (1.065 – 3.777) | 0.031 | 1.706 (0.798 – 3.644) | 0.168 |
| Perineural invasion (Present vs Absent) | 4.750 (2.413 – 9.350) | < 0.001 | 1.616 (0.610 – 4.281) | 0.335 |
| Tumor location |  | 0.103 |  |  |
| Cardia | Ref |  |  |  |
| Body | 0.591 (0.196 – 1.784) | 0.351 |  |  |
| Antrum | 0.958 (0.393 – 2.333) | 0.924 |  |  |
| Whole | 2.364 (0.361 – 15.455) | 0.369 |  |  |
| Lauren type (Diffuse vs Intestinal /Mixed) | 1.457 (0.752 – 2.820) | 0.265 |  |  |
| T classification (T3/T4 vs T1/T2) | 6.448 (3.014 – 13.795) | < 0.001 | 1.909 (0.643 – 5.666) | 0.244 |
| Lymph node status (Yes vs No) | 6.017 (2.972 – 12.182) | < 0.001 | 1.968 (0.666 – 5.816) | 0.221 |
| TNM stage (III/IV vs I/II) | 5.714 (2.892 – 11.292) | < 0.001 | 1.681 (0.575 – 4.915) | 0.342 |
| VFA-lesser omentum (cm^2^) | 0.914 (0.870 – 0.961) | < 0.001 | 0.913 (0.836 – 0.997) | **0.042** |
| VFA-maximum tumor (cm^2^) | 0.989 (0.982 – 0.997) | 0.009 | 1.003 (0.989 – 1.016) | 0.695 |
| VFA-L3 (cm^2^) | 0.996 (0.990 – 1.001) | 0.146 |  |  |
| **External test cohort 1** |  |  |  |  |
| Age (years) | 1.005 (0.975 – 1.036) | 0.745 |  |  |
| BMI (kg/m^2^) | 0.926 (0.848– 1.011) | 0.085 | 1.037 (0.910 – 1.181) | 0.589 |
| Gender (Male vs Female) | 1.231 (0.611 – 2.480) | 0.560 |  |  |
| Diabetes (Yes vs No) | 1.462 (0.489 – 4.365) | 0.497 |  |  |
| Tumor size (≥4cm vs <4cm) | 2.506 (1.345 – 4.666) | 0.004 | 1.277 (0.586 – 2.785) | 0.539 |
| Differentiation (Poorly/Undifferentiated vs Well/Moderately) | 1.255 (0.676 – 2.329) | 0.472 |  |  |
| Perineural invasion (Present vs Absent) | 2.819 (1.302 – 6.104) | 0.009 | 1.341 (0.526 – 3.418) | 0.539 |
| Tumor location |  | 0.015 |  | **0.048** |
| Cardia | Ref |  | Ref |  |
| Body | 0.186 (0.053 – 0.649) | 0.008 | 0.171 (0.040 – 0.721) | 0.016 |
| Antrum | 0.228 (0.067 – 0.778) | 0.018 | 0.613 (0.037 – 0.711) | **< 0.001** |
| Whole | 1.091 (0.153 – 7.802) | 0.931 | 0.634 (0.071 – 5.670) | 0.683 |
| Lauren type (Intestinal vs Diffuse/Mixed) | 0.973 (0.523 – 1.808) | 0.930 |  |  |
| T classification (T3/T4 vs T1/T2) | 4.364 (2.135 – 8.921) | < 0.001 | 0.965 (0.333 – 2.794) | 0.947 |
| Lymph node status (Yes vs No) | 5.749 (2.808 – 11.769) | < 0.001 | 2.124 (0.728 – 6.196) | 0.168 |
| TNM stage (III/IV vs I/II) | 5.363 (2.783 – 10.336) | < 0.001 | 3.499 (1.194 – 10.252) | **0.022** |
| VFA-lesser omentum (cm^2^) | 0.910 (0.864 – 0.958) | < 0.001 | 0.902 (0.819 – 0.993) | **0.035** |
| VFA-maximum tumor (cm^2^) | 0.992 (0.985 – 1.000) | 0.042 | 1.009 (0.994 – 1.025) | 0.249 |
| VFA-L3 (cm^2^) | 0.999 (0.994 – 1.004) | 0.693 |  |  |
| **External test cohort 2** |  |  |  |  |
| Age (years) | 1.007 (0.977 – 1.038) | 0.631 |  |  |
| BMI (kg/m^2^) | 0.852 (0.752– 0.966) | 0.012 | 1.145 (0.928 – 1.413) | 0.207 |
| Gender (Male vs Female) | 1.053 (0.548 – 2.023) | 0.876 |  |  |
| Diabetes (Yes vs No) | 0.712 (0.318 – 1.596) | 0.410 |  |  |
| Tumor size (≥4cm vs <4cm) | 2.096 (1.161 – 3.784) | 0.014 | 1.997 (0.782 – 5.104) | 0.148 |
| Differentiation (Poorly/Undifferentiated vs Well/Moderately) | 0.854 (0.465 – 1.568) | 0.611 |  |  |
| Perineural invasion (Present vs Absent) | 4.359 (2.250 – 8.444) | < 0.001 | 1.926 (0.675 – 5.500) | 0.221 |
| Tumor location |  | 0.073 |  | 0.172 |
| Cardia | Ref |  | Ref |  |
| Body | 0.492 (0.102 – 2.369) | 0.377 | 5.269 (0.628 – 44.197) | 0.126 |
| Antrum | 0.248 (0.053 – 1.154) | 0.075 | 1.807 (4.688 – 13.036) | 0.557 |
| Whole | 0.538 (0.062 – 4.691) | 0.575 | 2.046 (4.310 – 35.067) | 0.621 |
| Lauren type (Intestinal vs Diffuse/Mixed) | 0.811 (0.435 – 1.512) | 0.510 |  |  |
| T classification (T3/T4 vs T1/T2) | 1.360 (0.727 – 2.546) | 0.336 |  |  |
| Lymph node status (Yes vs No) | 1.546 (1.205 – 1.984) | 0.001 | 1.985 (1.035 – 3.806) | **0.039** |
| TNM stage (III/IV vs I/II) | 1.778 (0.975 – 3.242) | 0.060 | 0.347 (0.064 – 1.883) | 0.220 |
| VFA-lesser omentum (cm^2^) | 0.877 (0.840 – 0.916) | < 0.001 | 0.887 (0.814 – 0.966) | **0.006** |
| VFA-maximum tumor (cm^2^) | 0.981 (0.974 – 0.989) | < 0.001 | 0.998 (0.985 – 1.012) | 0.791 |
| VFA-L3 (cm^2^) | 0.993 (0.983 – 1.003) | 0.153 |  |  |

LVI, lymphovascular invasion; BMI, body mass index; VFA, visceral fat area; L3, the third lumbar vertebra.

**Table S4.** Logistic regression analysis of PNI in gastric cancer.

| **Variables** | | **Univariate Analysis**  **Odds ratio (95% CI) P** | | | **Multivariate Analysis**  **Odds ratio (95% CI) P** | | |
| --- | --- | --- | --- | --- | --- | --- | --- |
| **Training cohort** |  | | |  |  | |  |
| Age (years) | | 1.001 (0.981 – 1.021) | 0.937 | |  |  | |
| BMI (kg/m^2^) | | 0.874 (0.815– 0.938) | < 0.001 | | 0.949 (0.850 – 1.059) | 0.348 | |
| Gender (Male vs Female) | | 0.893 (0.574 – 1.388) | 0.615 | |  |  | |
| Diabetes (Yes vs No) | | 0.449 (0.234 – 0.862) | 0.016 | | 0.371 (0.160 – 0.861) | **0.021** | |
| Tumor size (≥4cm vs <4cm) | | 3.551 (2.303 – 5.475) | < 0.001 | | 1.099 (0.613 – 1.972) | 0.751 | |
| Differentiation (Poorly/Undifferentiated vs Well/Moderately) | | 2.029 (1.341 – 3.070) | 0.001 | | 1.323 (0.773 – 2.265) | 0.308 | |
| Lymphovascular invasion (Present vs Absent) | | 4.718 (3.036 –7.329) | < 0.001 | | 2.242 (1.296 – 3.878) | **0.004** | |
| Tumor location | |  | 0.242 | |  |  | |
| Cardia | | Ref |  | |  |  | |
| Body | | 0.893 (0.461 – 1.728) | 0.737 | |  |  | |
| Antrum | | 0.957 (0.538 – 1.704) | 0.882 | |  |  | |
| Whole | | 7.857 (0.952 – 64.838) | 0.056 | |  |  | |
| Lauren type (Intestinal vs Diffuse/Mixed) | | 1.140 (0.747 – 1.739) | 0.544 | |  |  | |
| T classification (T3/T4 vs T1/T2) | | 12.633 (7.620 – 20.944) | < 0.001 | | 5.962 (3.078 – 11.548) | **< 0.001** | |
| Lymph node status (Yes vs No) | | 6.053 (3.855 – 9.505) | < 0.001 | | 1.558 (0.768 – 3.158) | 0.219 | |
| TNM stage (III/IV vs I/II) | | 6.413 (4.020 – 10.230) | < 0.001 | | 1.367 (0.650 – 2.874) | 0.410 | |
| VFA-lesser omentum (cm^2^) | | 0.920 (0.892 – 0.948) | < 0.001 | | 0.933 (0.878 – 0.990) | **0.023** | |
| VFA-maximum tumor (cm^2^) | | 0.989 (0.984 – 0.994) | < 0.001 | | 1.004 (0.994 – 1.014) | 0.417 | |
| VFA-L3 (cm^2^) | | 0.997 (0.993 – 1.001) | 0.132 | |  |  | |
| **Internal validation cohort** | |  |  | |  |  | |
| Age (years) | | 0.988 (0.954 – 1.024) | 0.510 | |  |  | |
| BMI (kg/m^2^) | | 0.989 (0.907 – 1.079) | 0.804 | |  |  | |
| Gender (Male vs Female) | | 0.879 (0.445 – 1.738) | 0.711 | |  |  | |
| Diabetes (Yes vs No) | | 3.558 (0.744 – 17.015) | 0.112 | |  |  | |
| Tumor size (≥4cm vs <4cm) | | 1.838 (0.976 – 3.462) | 0.059 | | 0.499 (0.183 – 1.360) | 0.174 | |
| Differentiation (Poorly/Undifferentiated vs Well/Moderately) | | 1.859 (0.987 – 3.502) | 0.055 | | 1.267 (0.543 – 2.957) | 0.585 | |
| Lymphovascular invasion (Present vs Absent) | | 3.273 (1.708 – 6.272) | < 0.001 | | 1.173 (0.484 – 2.843) | 0.724 | |
| Tumor location | |  | 0.005 | |  | 0.095 | |
| Cardia | | Ref |  | | Ref |  | |
| Body | | 0.172 (0.055 – 0.538) | 0.002 | | 0.157 (0.037 – 0.666) | 0.012 | |
| Antrum | | 0.224 (0.078 – 0.641) | 0.005 | | 0.275 (0.072 – 1.046) | 0.058 | |
| Whole | | 1.667 (0.169 – 16.479) | 0.662 | | 0.333 (0.026 – 4.327) | 0.401 | |
| Lauren type (Intestinal vs Diffuse/Mixed) | | 1.519 (0.794 – 2.909) | 0.207 | |  |  | |
| T classification (T3/T4 vs T1/T2) | | 13.510 (5.860 – 31.145) | < 0.001 | | 11.391 (3.158 – 41.086) | **< 0.001** | |
| Lymph node status (Yes vs No) | | 5.285 (2.684 – 10.404) | < 0.001 | | 0.786 (0.220 – 2.812) | 0.711 | |
| TNM stage (III/IV vs I/II) | | 5.291 (2.644 – 10.587) | < 0.001 | | 1.845 (0.572 – 5.957) | 0.305 | |
| VFA-maximum tumor (cm^2^) | | 0.988 (0.980 – 0.996) | 0.003 | | 0.998 (0.983 – 1.013) | 0.759 | |
| VFA-lesser omentum (cm^2^) | | 0.899 (0.856 – 0.945) | < 0.001 | | 0.914 (0.841 – 0.994) | **0.037** | |
| VFA-L3 (cm^2^) | | 0.996 (0.990 – 1.001) | 0.144 | |  |  | |
| **External test cohort 1** | |  |  | |  |  | |
| Age (years) | | 0.985 (0.942 – 1.030) | 0.501 | |  |  | |
| BMI (kg/m^2^) | | 0.951 (0.862– 1.048) | 0.310 | |  |  | |
| Gender (Male vs Female) | | 1.682 (0.739 – 3.825) | 0.215 | |  |  | |
| Diabetes (Yes vs No) | | 2.510 (0.783 – 8.044) | 0.122 | |  |  | |
| Tumor size (≥4cm vs <4cm) | | 3.091 (1.390 – 6.874) | 0.006 | | 1.441 (0.592 – 3.507) | 0.421 | |
| Differentiation (Poorly/Undifferentiated vs Well/Moderately) | | 0.986 (0.456 – 2.131) | 0.971 | |  |  | |
| Lymphovascular invasion (Present vs Absent) | | 2.819 (1.302 – 6.104) | 0.009 | | 1.156 (0.468 – 2.858) | 0.753 | |
| Tumor location | |  | 0.562 | |  |  | |
| Cardia | | Ref |  | |  |  | |
| Body | | 1.685 (0.340 – 8.351) | 0.523 | |  |  | |
| Antrum | | 1.434 (0.292 – 7.033) | 0.657 | |  |  | |
| Whole | | 3.900 (0.494 – 30.758) | 0.196 | |  |  | |
| Lauren type (Intestinal vs Diffuse/Mixed) | | 1.014 (0.469 – 2.193) | 0.971 | |  |  | |
| T classification (T3/T4 vs T1/T2) | | 26.231 (3.489 – 197.182) | 0.002 | | 15.836 (1.783 – 140.632) | **0.013** | |
| Lymph node status (Yes vs No) | | 3.156 (1.289 – 7.727) | 0.012 | | 0.353 (0.074 – 1.690) | 0.192 | |
| TNM stage (III/IV vs I/II) | | 4.560 (1.978 – 10.509) | < 0.001 | | 3.196 (0.788 – 12.965) | 0.104 | |
| VFA-lesser omentum (cm^2^) | | 0.890 (0.826 – 0.958) | 0.002 | | 0.878 (0.775 – 0.995) | **0.041** | |
| VFA-maximum tumor (cm^2^) | | 0.989 (0.978 – 0.999) | 0.031 | | 1.007 (0.990 – 1.024) | 0.409 | |
| VFA-L3 (cm^2^) | | 0.999 (0.993 – 1.005) | 0.755 | |  |  | |
| **External test cohort 2** | |  |  | |  |  | |
| Age (years) | | 0.976 (0.949 – 1.005) | 0.103 | |  |  | |
| BMI (kg/m^2^) | | 0.760 (0.664 – 0.869) | < 0.001 | | 0.899 (0.757 – 1.067) | 0.224 | |
| Gender (Male vs Female) | | 1.207 (0.658 – 2.215) | 0.543 | |  |  | |
| Diabetes (Yes vs No) | | 0.861 (0.396 – 1.875) | 0.707 | |  |  | |
| Tumor size (≥4cm vs <4cm) | | 2.026 (1.169 – 3.512) | 0.012 | | 1.488 (0.624 – 3.547) | 0.370 | |
| Differentiation (Poorly/Undifferentiated vs Well/Moderately) | | 1.049 (0.597 – 1.844) | 0.867 | |  |  | |
| Lymphovascular invasion (Present vs Absent) | | 4.359 (2.250 – 8.444) | < 0.001 | | 1.947 (0.706 – 5.370) | 0.198 | |
| Tumor location | |  | 0.648 | |  |  | |
| Cardia | | Ref |  | |  |  | |
| Body | | 0.578 (0.189 – 1.768) | 0.336 | |  |  | |
| Antrum | | 0.522 (0.174 – 1.571) | 0.248 | |  |  | |
| Whole | | 0.833 (0.157 – 4.436) | 0.831 | |  |  | |
| Lauren type (Intestinal vs Diffuse/Mixed) | | 1.027 (0.574 – 1.836) | 0.929 | |  |  | |
| T classification (T3/T4 vs T1/T2) | | 2.389 (1.287 – 4.435) | 0.006 | | 1.156 (0.390 – 3.427) | 0.793 | |
| Lymph node status (Yes vs No) | | 1.390 (1.117 – 1.729) | 0.003 | | 0.772 (0.441 – 1.352) | 0.365 | |
| TNM stage (III/IV vs I/II) | | 2.297 (1.327 – 3.978) | 0.003 | | 2.896 (0.593 – 14.142) | 0.189 | |
| VFA-lesser omentum (cm^2^) | | 0.864 (0.824 – 0.906) | < 0.001 | | 0.907 (0.825 – 0.996) | **0.041** | |
| VFA-maximum tumor (cm^2^) | | 0.975 (0.966 – 0.983) | < 0.001 | | 0.994 (0.978 – 1.010) | 0.471 | |
| VFA-L3 (cm^2^) | | 0.993 (0.985 – 1.002) | 0.119 | |  |  | |

PNI, perineural invasion; BMI, body mass index; VFA, visceral fat area; L3, the third lumbar vertebra

**Table S5.** Baseline characteristics of patients according to Low and High VFA in training cohort.

| **Variables** | **Training cohort**  **(N = 389)** | | |
| --- | --- | --- | --- |
|  | **Low VFA**  **(*N* = 242)** | **High VFA**  **(*N* = 147)** | ***P* value** |
| Age (years) | 58.72±9.7 | 61.03±8.4 | 0.018 |
| BMI (kg/m^2^) | 21.8±2.8 | 24.5±2.9 | <0.001 |
| Gender |  |  | 0.048 |
| Male | 167 (69.0) | 115 (78.2) |  |
| Female | 75 (31.0) | 32 (21.8) |  |
| Smoking |  |  | 0.585 |
| Yes | 132 (54.5) | 76 (51.7) |  |
| No | 110 (45.5) | 71 (48.3) |  |
| Drinking |  |  | 0.546 |
| Yes | 80 (33.1) | 53 (36.1) |  |
| No | 162 (66.9) | 94 (63.9) |  |
| Hypertension |  |  | 0.112 |
| No | 209 (86.4) | 118 (80.3) |  |
| Yes | 33 (13.6) | 29 (19.7) |  |
| Diabetes |  |  | 0.113 |
| No | 224 (92.6) | 129 (87.8) |  |
| Yes | 18 (7.4) | 18 (12.2) |  |
| Tumor size (cm) |  |  | 0.148 |
| < 4 | 125 (51.7) | 87 (59.2) |  |
| ≥ 4 | 117 (48.3) | 60 (40.8) |  |
| Differentiation |  |  | 0.095 |
| Well/Moderately | 88 (36.4) | 66 (44.9) |  |
| Poorly/Undifferentiated | 154 (63.6) | 81 (55.1) |  |
| Lymphovascular invasion |  |  | 0.236 |
| Negative | 120 (49.6) | 82 (55.8) |  |
| Positive | 122 (50.4) | 65 (44.2) |  |
| Perineural invasion |  |  | 0.006 |
| Negative | 83 (34.3) | 71 (48.3) |  |
| Positive | 159 (65.7) | 76 (51.7) |  |
| Location |  |  | 0.137 |
| Cardia | 45 (18.6) | 17 (11.6) |  |
| Body | 57 (23.6) | 32 (21.8) |  |
| Antrum | 129 (53.3) | 94 (63.9) |  |
| Whole stomach | 11 (4.5) | 4 (2.7) |  |
| Surgical procedure |  |  | 0.027 |
| Partial gastrectomy | 183 (75.6) | 125 (85.0) |  |
| Total gastrectomy | 59 (24.4) | 22 (15.0) |  |
| D2 lymph node dissection |  |  | 0.001 |
| Yes | 196 (81.0) | 96 (65.3) |  |
| No | 46 (19.0) | 51 (34.7) |  |
| Lauren type |  |  | 0.482 |
| Diffuse | 90 (37.2) | 50 (34.0) |  |
| Intestinal | 97 (40.1) | 68 (46.3) |  |
| Mixed | 55 (22.7) | 29 (19.7) |  |
| Depth of invasion |  |  | <0.001 |
| T1/T2 | 59 (24.4) | 65 (44.2) |  |
| T3/T4 | 183 (75.6) | 82 (55.8) |  |
| Lymph node metastasis |  |  | 0.016 |
| Yes | 166 (68.6) | 83 (56.5) |  |
| No | 76 (31.4) | 64 (43.5) |  |
| pTNM stage |  |  | <0.001 |
| I/ II | 112 (46.3) | 101 (68.7) |  |
| III | 130 (53.7) | 46 (31.3) |  |
| Adjuvant chemotherapy |  |  | 0.001 |
| Yes | 162 (66.9) | 74 (50.3) |  |
| No | 80 (33.1) | 73 (49.7) |  |
| Borrmann type |  |  | 0.088 |
| Type 1 | 7 (2.9) | 7 (4.8) |  |
| Type 2 | 44 (18.2) | 31 (21.1) |  |
| Type 3 | 167 (69.0) | 104 (70.7) |  |
| Type 4 | 24 (9.9) | 5 (3.4) |  |
| CEA |  |  | 0.729 |
| Normal | 206 (85.1) | 127 (86.4) |  |
| Elevated | 36 (14.9) | 20 (13.6) |  |
| CA199 |  |  | 0.030 |
| Normal | 206 (85.1) | 136 (92.5) |  |
| Elevated | 36 (14.9) | 11 (7.5) |  |
| CA724 |  |  | 0.665 |
| Normal | 14 (5.8) | 7 (4.8) |  |
| Elevated | 228 (94.2) | 140 (95.2) |  |
| Albumin (g/L) | 39.6±5.2 | 39.6±4.6 | 0.924 |
| White blood cell (**10^9** /L) | 6.44±1.99 | 6.48±1.90 | 0.831 |
| Platelet (**10^9** /L) | 255.1±80.6 | 240.8±78.9 | 0.089 |
| Hemoglobin (g/L) | 129.9±22.9 | 134.5±23.2 | 0.087 |

BMI, body mass index; CEA, carcinoembryonic antigen; CA199, carbohydrate antigen 19-9; CA724, carbohydrate antigen 724; VFA, visceral fat area;

L3, the third lumbar vertebra.
